# Interfacial Bonding Energy on the Interface between ZChSnSb/Sn Alloy Layer and Steel Body at Microscale

**DOI:** 10.3390/ma10101128

**Published:** 2017-09-25

**Authors:** Jianmei Wang, Quanzhi Xia, Yang Ma, Fanning Meng, Yinan Liang, Zhixiong Li

**Affiliations:** 1 Engineering Research Center Heavy Machinery Ministry of Education, Taiyuan University of Science and Technology, Taiyuan 030024, China; 18335100976@163.com (Q.X.); tilit2017@163.com (Y.M.); mfn0857@163.com (F.M.); 13935124901@163.com (Y.L.); 2 School of Mechatronic Engineering, China University of Mining Technology, Xuzhou 221116, China; zhixiongli@whut.edu.cn; 3 School of Manufacturing & Mechanical Engineering, UNSW, Sydney 2200, Australia

**Keywords:** molecular dynamics, Sn layer, interfacial bonding performances, bonding strength, Babbitt layer thickness

## Abstract

To investigate the performance of bonding on the interface between ZChSnSb/Sn and steel body, the interfacial bonding energy on the interface of a ZChSnSb/Sn alloy layer and the steel body with or without Sn as an intermediate layer was calculated under the same loadcase using the molecular dynamics simulation software Materials Studio by ACCELRYS, and the interfacial bonding energy under different Babbitt thicknesses was compared. The results show that the bonding energy of the interface with Sn as an intermediate layer is 10% larger than that of the interface without a Sn layer. The interfacial bonding performances of Babbitt and the steel body with Sn as an intermediate layer are better than those of an interface without a Sn layer. When the thickness of the Babbitt layer of bushing is 17.143 Å, the interfacial bonding energy reaches the maximum, and the interfacial bonding performance is optimum. These findings illustrate the bonding mechanism of the interfacial structure from the molecular level so as to ensure the good bonding properties of the interface, which provides a reference for the improvement of the bush manufacturing process from the microscopic point of view.

## 1. Introduction

Due to its small friction coefficient, low loss, and high rigidity, oil-film bearing is widely used in the steel, mine, metallurgy, and electric power professions [1]. It is mainly used in heavy rolling mill equipment [2]. Oil-film bearing is an important part of rolling mill equipment; its most important component is bushing. The interfacial bonding performance on the interface between the Babbitt and steel of bushing is related to the performance of oil-film bearing and ultimately affects the safety and the reliability of operation of the production line [3].

With the significant improvement of computer performance, the study of molecular dynamics simulations has been well developed. Molecular dynamics modeling can be performed with the calculation of atoms or molecules at the micro scale, and simulation has been widely used in composite materials. Guo et al. [4] used a molecular dynamics method to simulate the interactions between a Si/Al composite material and a Ni coating surface. The equilibrium interfacial atomic configuration was obtained after molecular dynamics optimization, and the interaction energy was calculated. Luo et al. [5] performed molecular dynamics simulations on an Al/SiC interface and calculated the bonding energy of fifteen kinds of interface atomic configurations formed by three low-index surfaces of Al and SiC. Mo et al. [6] simulated three composite materials involving graphite nanosheets (NanoG)/AgCl/polymer at the atomic scale and the mesoscale using a molecular dynamics method and analyzed the interface energy and structure.

Du et al. [7] applied molecular dynamics to construct an interface structure between SU-8(a photoresist for high-aspect-ratio and 3D submicron lithography materials) photoresist and Ni substrate, and studied the influence of post-drying temperature on interfacial bonding. Yuan [8] used a molecular dynamics method to simulate the tension in the deformation behavior of different grain sizes of polycrystalline silver nanowires and made detailed a analysis of the grain size of polycrystalline silver nanowires with elastic moduluses, yield strengths, and influences on the plastic deformation mechanism. Melenev et al. [9] studied magnetic nanoparticles in a fluid environment using kinetics and the molecular lattice Boltzmann method and found that a weak magnetic dipole interaction between objects leads to the decrease of relaxation time and that the diffusion degree of acceleration increased with magnetic force. Chen [10] used spectral analysis and NMR (Nuclear Magnetic Resonance) techniques to study the synthesis and properties of novel fluorinated anionic surfactants; the experimental results show that SPBS (novel fluorinated anionic surfactant) has excellent surface activity and bonding properties. The above literature review displays that molecular dynamics has its unique advantages in the analysis of composite interface problems. Domestic and international experts have done some relevant research on the performance of the Babbitt layer of oil-film bearing bushing.

Zhang et al. [11] observed the changes to the surface friction sliding speed by laser ablation of the Babbitt alloy matrix and made a fluid dynamics simulation of the surface pressure distribution. Kamal [12] studied the influencing factors of the rapid solidification of the Babbitt on the mechanical properties and concluded that boundary slip is the possible mechanism of ambient temperature creep in melt spinning. However, there is less literature at home and abroad on the bonding properties of Babbitt alloy and the steel body of oil-film bearing bushing. Therefore, it is necessary to make a thorough analysis of the bonding properties of Babbitt and a steel body.

In this study, with Sn-based Babbitt alloy bushing as the research object, the simulation analysis, with or without Sn as an intermediate layer, was done using a molecular dynamics simulation, and the conditions to produce the maximum bonding energy were obtained. The Sn layer plays a transitional role in alleviating the stress concentrated between the steel body and the alloy layer, reducing the mutation of the physical properties of the interface, as well as improving the bonding performances between the Babbitt layer and the steel body. From a microscopic point of view, the atomic diffusion thickness of the interfacial structure of the Sn layer is a bit larger. Sn ions in the Sn layer have more absorption capability than Sn atoms in the Babbitt layer and iron atoms in the steel body. The mechanism of interfacial bonding at the micro scale was investigated, and the bonding properties of interface structures with different thicknesses of Babbitt layer were studied. The study of the Sn layer has a reference value for the bonding properties of materials.

## 2. Fundamentals of Molecular Dynamics

Molecular dynamics mainly focuses on multi-body problems at the microscopic scale, modeling and calculation at the atomic or molecular level can better forecast the properties of material dynamics, and the simulation accuracy lies in the selection of the potential function of the atomic interaction within the system. In a polyatomic system, the different positions of an atom will affect the effective interactions among other atoms. In this study, multi-body potential energy is used to accurately represent the potential energy of a polyatomic system [13].

The molecular dynamics simulation at the equilibrium state is carried out under a certain ensemble. Here, the canonical ensemble is used. The atomic number, N, the volume, V, and the temperature, T, are kept constant, and the total momentum is zero. Considering that the kinetic system contains N molecules or atoms, the systematic energy is the sum of the total kinetic energy of the molecules and the total potential energy of the system. The total potential energy is a function of the positions of the atoms or molecules $U\left({\vec{r}}_{1},{\vec{r}}_{2},\cdots ,{\vec{r}}_{n}\right)$, and it generally consists of the intermolecular potential energy between atoms of non-bonded Van Der Waals action potential (VDW) and the inner potential energy within the molecules (int), i.e.,

(1) $$U=U_{\mathrm{VDW}}+U_{\mathrm{int}}$$

Generally, Van Der Waals’s function can be approximated by the addition of a Van Edward Mars effect to the atoms.

(2) $$\begin{array}{l} U_{\mathrm{VDW}}=U_{12}+U_{13}+\cdots +U_{1n}+U_{23}+U_{24}\cdots \\ \;=\;\sum_{i=1}^{n-1}\sum_{j=i+1}^{n}U_{\mathrm{ij}}(r_{\mathrm{ij}}) \end{array}$$

where $r_{\mathrm{ij}}$ is the distance between the jth and ith atom.

The intramolecular potential energy is the sum of the internal coordinate potential energy of each type.

According to classical mechanics, the force acting on any ith atom in the system is the gradient of potential energy.

(3) $${\vec{F}}_{i}=-\nabla_{i}U=-(\vec{i}\frac{\partial }{\partial_{X_{i}}}+\vec{j}\frac{\partial }{\partial_{Y_{i}}}+\vec{k}\frac{\partial }{\partial_{Z_{i}}})U$$

In turn, the acceleration of the ith atom is obtained by Newton’s Laws of Motion:

(4) $${\vec{a}}_{i}=\frac{{\vec{F}}_{i}}{m_{i}}$$

Integrating Newton’s laws of motion and equations with time can allow the prediction of the velocity and position of the ith atom after time t.

(5) $$\left\{ \begin{array}{l} \frac{d^{2}}{dt^{2}}{\vec{r}}_{i}=\frac{d}{dt}{\vec{v}}_{i}={\vec{a}}_{i}\\ \;{\vec{v}}_{i}={\vec{v}}_{i}^{0}+{\vec{a}}_{i}t\\ \;{\vec{r}}_{i}={\vec{r}}_{i}^{0}+{\vec{v}}_{i}^{0}t+\frac{1}{2}{\vec{a}}_{i}t^{2} \end{array}\right.$$

where $\vec{r}$ and $\vec{v}$ respectively present the position and velocity of particles, with the superscript ‘0’ as the initial value of each physical quantity.

The basic principle of molecular dynamics is to calculate the potential energy of a system and the force and acceleration of each atom in the system using Newton’s Law of Motion; then, letting t=δt, the position and speed of each molecule can be obtained. In this way, the position, velocity, and acceleration of the mid molecular motion of the system can be obtained, and the positions of the molecules at different time is called the trajectory. During the whole process, the total systematic energy remains unchanged, but the molecular internal energy and the kinetic energy continuously transform into each other, thus changing the temperature of the system, and the system will traverse each point on the potential energy surface. The current potential energy of the system is accumulated by sampling from each state of the system, and then the configuration integral is calculated.

## 3. Interface Simulations with and without Sn Layer

The interface structure for the bushing with or without a Sn layer was built using the molecular dynamics software Materials Studio, and the molecular dynamics simulations were performed. Meanwhile, the interfacial bonding energy of different interface structures can be calculated; the optimum function with or without a Sn layer was found so as to produce the best bonding performance of the bushing; and the influence of the Sn layer on the bonding performance of the bushing was analyzed.

### 3.1. Establishment of Model

When constructing the interface structures of the model under different conditions, some parameters of the crystal structure need to be determined, including the space group of the crystal, the parameters of the crystal lattices, the atomic coordinates within the crystal, etc. The relevant parameters in this article, as shown in Table 1, can be obtained from the database ICSD (Inorganic Crystal Structure Database).

**Figure 1:**
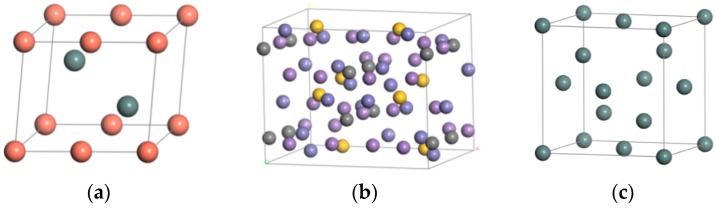
The crystal structure of the raw materials. (**a**) Babbitt cell structure; (**b**) Steel cell structure; (**c**) Sn crystal structure.

First of all, according to the parameters of the material cells, the unit cell is constructed using the Build crystals software tool, and the atoms are added on the basis of the atomic coordinates. The original crystal structures of steel and Babbitt were constructed. The original crystal structure of Sn was imported from the software library. Figure 1 shows the crystal structure of Babbitt, steel body, and Sn. Two crystal structures in Figure 1a,b are constructed by the Materials Visualizer module in the Materials software, and the cell structures of the Babbitt and the steel body are obtained by adding atoms according to the coordinates of the atoms.

Then, the tangent plane is created on the constructed lattice structure. The general section principle is to shear the low index surface, on which the surface has the lowest interfacial energy. A simple tangent plane of each cell was obtained using the Cleave Surface tool. The original cells of the steel body, Sn, and Babbitt were tangential along the (100) surface. In order to produce approximate sizes during the construction of the interface and avoid larger error, the steel body cell was extended into a 2 × 2 × 1 super-cell, the Sn cell was extended into a 3 × 3 × 1 super-cell, and the Babbitt cell was extended into a 6 × 5 × 1 super-cell.

The super cell structure was respectively constructed by turning a 2D structure into 3D structure using the Build Vacuum Slab tool, selecting the appropriate thickness and setting the thickness of the vacuum layer as 3 Å. The force field was distributed across all the constructed supercell structures of the materials [14]. The force field was assigned to all atoms in the cell using the Typing function. Each atom in the crystal cell was distributed within the force field using the COMPASS force field tool [15], and the corresponding force field was chosen for each atoms. Then the force field of atoms was distributed.

Vander Ed Ley and Coulomb forces need to be considered during optimization, with the Minimizer function operating on cell surface structure for the distribution of the stress field of energy minimization. The surface structure was optimized, and the optimization of the atomic position became more accurate. The original atoms before optimization need to be fixed at the bottom of the cell structure. In order to achieve surface energy optimization, only the atoms on the surface layers were optimized. The minimizer system structure is improved so as to achieve the most stable configuration [16]. The wave energy in the subsequent dynamics simulation became smaller, and the convergence time became shorter. In order to reduce the amount of calculation, the atom-based calculation method is chosen, and the default value of the cutoff distance is 9.5 Å.

**Figure 2:**
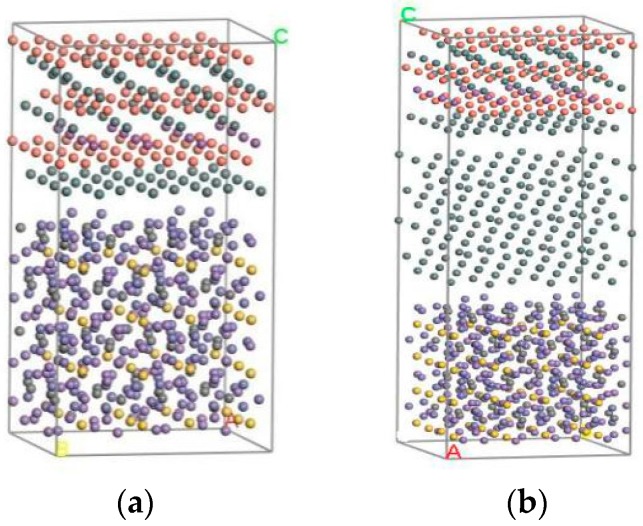
The interface structure of the molecular model. (**a**) Without a Sn interface structure; (**b**) With a Sn interface structure.

Finally, the Build layer tool was used to construct interface structures under different conditions. The Babbitt layer and steel layer on the optimized surface formed a two-layer material interface structure in a box. The optimization of the Babbitt layer, steel layer, and Sn layer cell constructed a three-layer material interface structure. After adjusting the direction of crystal structure, the corresponding surfaces after optimization are constructed as two-layer and three-layer material interface structures, as shown in Figure 2. Figure 2a gives the cell structure of the two-layer interface between the steel body and the Babbitt, and Figure 2b gives the three-layer interface structure of the steel body, the Sn layer, and the Babbitt.

### 3.2. Analogy Method

All simulations are carried out in the Discover module. When optimizing the surface, the Smart Minimizer was selected for energy optimization. Considering the computation volume and the calculating speed, the level of convergence chooses ‘Fine’ and the maximum number of iteration steps was 10,000 steps. Before the dynamics calculation, several layers of atoms far from the interface were fixed because only a small number of atoms on the upper layer will interact with each other [17].

**Figure 3:**
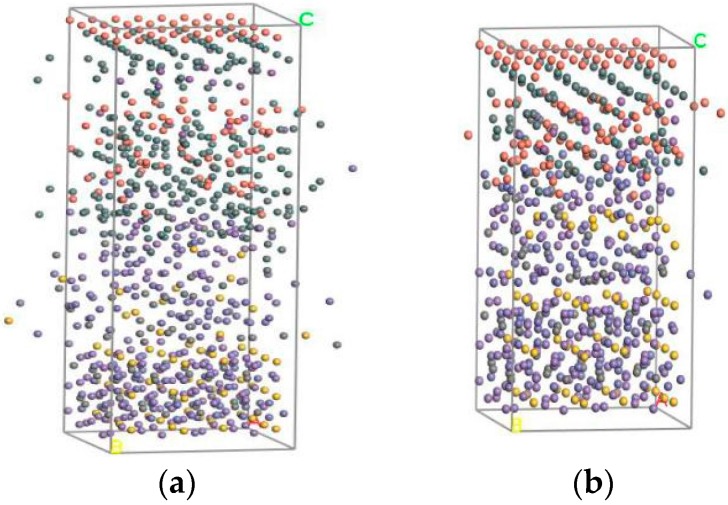
The interface molecular equilibrium structure. (**a**) Without a Sn interface balance structure; (**b**) With a Sn interface balance structure.

Molecular Dynamics uses a canonical ensemble (NVT) with constant atoms (N), constant volume (V), and constant temperature (T). Derived from the literature [18], the temperature of the maximum bonding energy is 512 K, so the initial temperature is 512 K. The Andersen algorithm is chosen as the temperature control method; the simulation time and time step are the default values; and ‘full’ is chosen as the save mode of the trajectory form of molecular conformation. Figure 3 gives the balanced two-layer and three-layer interfacial molecular structures, which display the simulation status of the above models. The Smart Minimizer method is used to analyze the models, and the balanced equilibrium structure under molecular motion is obtained; then the interfacial binding energy can be obtained.

### 3.3. Calculation of Interfacial Bonding Energy

The bonding performance of the interface is usually evaluated from the interfacial bonding energy. If the interfacial energy is larger, the power needed to destroy the interface structure will be larger, so the s interfacial bonding is stronger. Here, the calculation method for the bonding energy proposed in the literature [19] and the formula for the interface structure of three-layer materials presented in literature [20] are integrated.

The interfacial bonding energy is defined as the interactive bonding energy of two-layer materials, i.e.,$E_{\mathrm{bonding}}=E_{\mathrm{interaction}}$. The calculation of the bonding energy of two-layer materials is based on the formula from the literature [17].

(6) $$E_{\mathrm{interaction}}=E_{\mathrm{total}}-(E_{\mathrm{steel}}+E_{\mathrm{Babbitt}})$$

Formula (6) is used for calculating two-layer materials. When calculating the three-layer materials involved in steel, Sn, and Babbitt, the formula cannot be used directly.

The following formula can be obtained through an analysis of the principle of bonding energy.

(7) $$E_{\mathrm{interaction}3}=E_{\mathrm{Sn}}+E_{\mathrm{interaction}1}+E_{\mathrm{interaction}2}$$

where $E_{\mathrm{interaction}3}$ is the total interaction energy of the three-layer structure interface, kcal/mol; $E_{\mathrm{Sn}}$ is the three-layer structure, removing the single point energy of steel and Babbitt alloys, kcal/mol; $E_{\mathrm{interaction}1}$ is the interaction energy of the two-layer structure of Babbitt and a Sn layer, kcal/mol; and $E_{\mathrm{interaction}2}$ is the interaction energy between the steel and the Sn layer, kcal/mol.

The formula for the two-layer interface structure can be obtained from Formula (8).

(8) $$E_{\mathrm{interaction}1}=E_{\mathrm{total}1}-(E_{\mathrm{Babbitt}}+E_{\mathrm{Sn}})$$

where $E_{\mathrm{total}1}$ is the total energy of the two-layer structure with Babbitt and Sn, kcal/mol.

(9) $$E_{\mathrm{interaction}2}=E_{\mathrm{total}2}-(E_{\mathrm{steel}}+E_{\mathrm{Sn}})$$

where $E_{\mathrm{total}2}$ is the total energy of the two-layer interface between steel and Sn, kcal/mol.

Formulas (8) and (9) are substituted into Formula (7).

(10) $$E_{\mathrm{interaction}3}=E_{\mathrm{total}1}+E_{\mathrm{total}2}-(E_{\mathrm{Babbitt}}+E_{\mathrm{steel}}+E_{\mathrm{Sn}})$$

By the following formula:

(11) $$E_{\mathrm{total}3}=E_{\mathrm{total}1}+E_{\mathrm{total}2}-E_{\mathrm{Sn}}$$

where $E_{\mathrm{total}3}$ is the total energy of the Babbitt layer, Sn layer, and steel layer, kcal/mol.

Derived from Formulas (10) and (11):

(12) $$E_{\mathrm{interaction}3}=E_{\mathrm{total}3}-(E_{\mathrm{Babbitt}}+E_{\mathrm{steel}})$$

As can be seen from Formula (12), the calculation formula for a three-layer structure of interfacial energy is the same as that for a two-layer interface structure. When calculating the interface structure of a three-layer material, Sn as an interlayer is regarded as the bonding interface layer between the steel layer and the Babbitt alloy layer. Therefore, when calculating the interfacial binding energies of two-layer materials and three-layer materials, the formula can be unified as below:

(13) $$E_{\mathrm{interaction}}=E_{\mathrm{total}}-(E_{\mathrm{steel}}+E_{\mathrm{Babbitt}})$$

where $E_{\mathrm{bonding}}$ is the bonding energy of the interface structure, kcal/mol; $E_{\mathrm{interaction}}$ is the interaction energy of multi-layer materials in the interface structure, kcal/mol; $E_{\mathrm{total}}$ is the total energy of the multi-layer materials in interface structures, kcal/mol; $E_{\mathrm{steel}}$ is the energy of the interfacial structure, removing the energy of the Sn layer and the Babbitt layer, kcal/mol; and $E_{\mathrm{Babbitt}}$ is the energy of the interface structure, removing the energy of the steel body and the Sn layer, kcal/mol.

From the above deduction:

(14) $$E_{\mathrm{interaction}}=E_{\mathrm{total}}-(E_{1}+E_{2})$$

where $E_{1}$ and $E_{2}$ are the energies of the materials on the boundary of the interface structure, kcal/mol. It can be extended to calculate the bonding energies of other interface structures with two or three layers [21].

The constraints on the atoms in the whole structure need to be removed during the calculation of the total energy. When calculating the energy of some materials, the atoms of the other materials in the interface structure need to be removed; then the calculation can be done.

### 3.4. Simulation Results

After the molecular dynamics simulation of the interface structure, the energy of the stable interface structure was calculated. The total energy of the two layers and the energy of each part of the material without the Sn layer are calculated. The total energy of the three layers and the energy of each part of the material in the interface structure with the Sn layer are obtained. The results are shown in Table 2.

The bonding energy of the interface with Sn is 570,428.82 kcal/mol, and the bonding energy of the interface without Sn is 519,938.92 kcal/mol. The results show that the bonding energy of the interface with Sn as intermediate layer is 10% larger than that without a Sn layer. For the energy analysis of different interfaces, the compositions of various types of interface energy are shown in Table 3.

**Figure 4:**
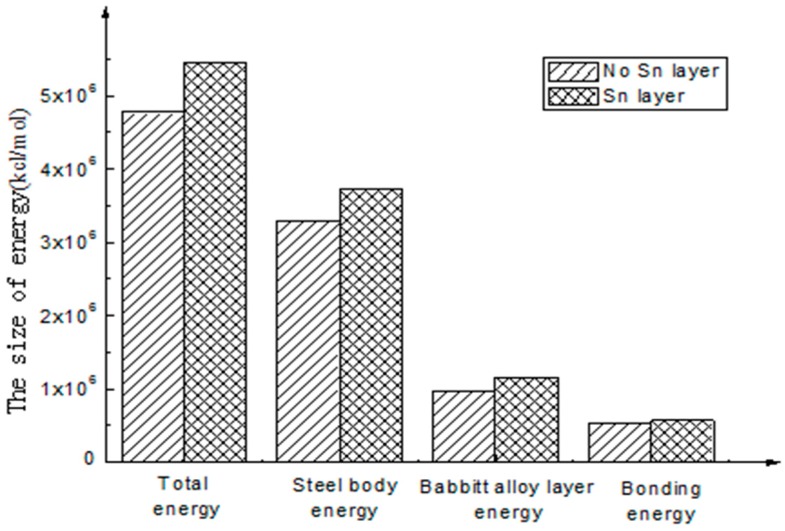
Interface energy composition.

**Figure 5:**
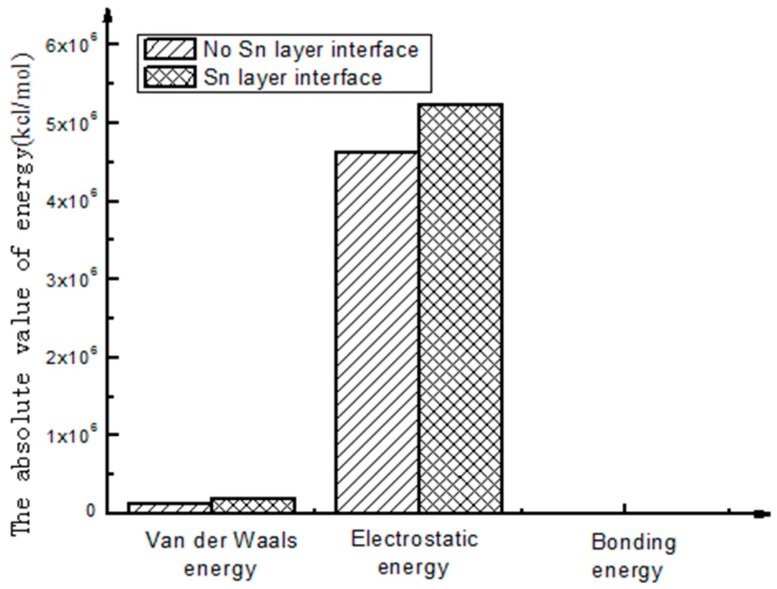
Energy of different interface structures.

To better intuitively view and compare these figures, a histogram is used to represent the above energy, as shown in Figure 4 and Figure 5.

As shown in Figure 5, through the analysis of the interfacial energy on the different interfaces using molecular dynamics, there exists an interaction between the energies of the molecules on the interfaces, and Van Der Waals energy and electrostatic energy are the main energy forms. The latter is much larger than the former. As to the final interfacial bonding energy, the main influencing factor is non-bonding energy. Compared with non-bonding energy, the bonding energy is small and seems to be approximate to zero.

(1) The calculation results show that the interfacial energy of the interface with Sn layer is slightly larger than that without a Sn layer. As shown in Figure 5, the bonding energy of the interface layer with the Sn layer is larger than that without a Sn layer by 10%. When there is a Sn layer, the bonding performance between steel and Babbitt alloy with a Sn layer is better than that without a Sn layer and is less easily destroyed. The results from the molecular dynamics simulation are consistent with those from the macroscopic law. A Sn layer can buffer the stress concentration due to the mutation of the compositions between the steel body and the lining layer, thus reducing interface mutation and producing better bonding performance between the Babbitt layer and the steel layer.
(2) As seen from the compositions of different interfacial energies, the main forces on an interface structure are Van Der Waals’ force and electrostatic force. The interaction energies among the interfaces are mainly Van Der Waals’ energy and electrostatic energy. Bonding energy can be ignored due to its small fraction. The main factor influencing the interfacial bonding energy is non-bonding energy.

## 4. Simulation on Interface with Different Babbitt Thickness

The lightweight nature of oil-film bearing and the reduction of Babbitt alloy thickness have been important directions for the improvement of the oil-film bearing process. Till now, there has been little literature about the influence of Babbitt thickness on bonding performances. The following section will focus on the effect of Babbitt thickness on bonding properties from the micro perspective.

### 4.1. Simulation Process

The cell structures of Babbitt and steel are respectively constructed according to the method in Section 3.1. By adjusting the thickness fraction of the Babbitt layer after cutting; different thicknesses of Babbitt layer are obtained. When setting the ‘Thickness’ option, the thickness of the Babbitt layer is variously set as 1.167, 1.833, 2.333, 2.833, and 3.167, which corresponds to the thickness fraction of the crystal structure, and the corresponding Babbitt thicknesses are 8.521, 13.391, 17.043, 20.695, and 23.13 Å. After entitling a force field and optimizing the alloy surface, the interface structures between steel and Babbitt with different thicknesses were constructed.

With the same simulation method as in Section 3.2, the energy of the balanced interface structure was calculated, and the energy calculation is done for each part of the interface structure with different Babbitt thicknesses. Formula (6) was used to calculate the interaction energy of the interface structure with different Babbitt thicknesses, and the bonding energies of different Babbitt thickness are obtained respectively [22].

### 4.2. Simulation Results

Through molecular dynamics simulation of the interface structure of Babbitt with different thicknesses, the energy of each stable interface structure was calculated respectively, and the energy of each part of different interfaces is obtained using the energy calculation function in the Discover module. The results are shown in Table 4.

By unit conversion, 1 kcal/mol = 4.180 KJ/mol; the bonding energies of the Babbitt layer with different thicknessess are listed in Table 5.

**Figure 6:**
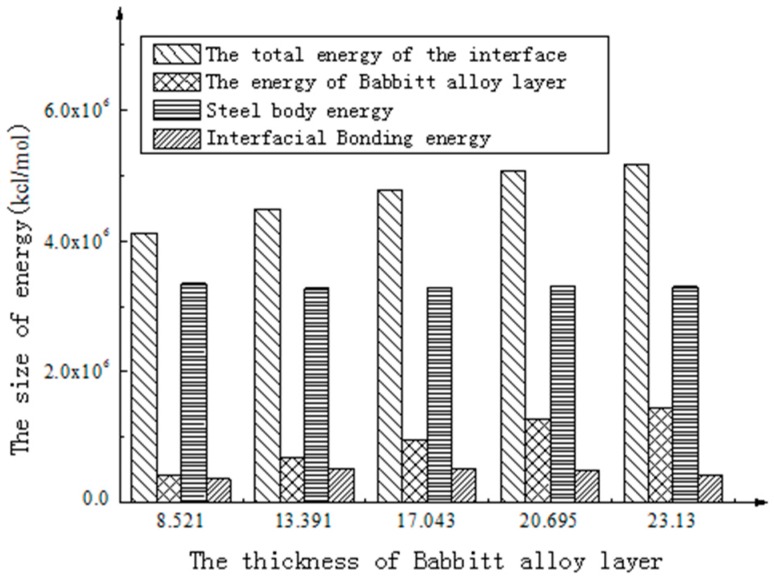
The energy on the interface of Babbitt alloy with different thicknesses.

**Figure 7:**
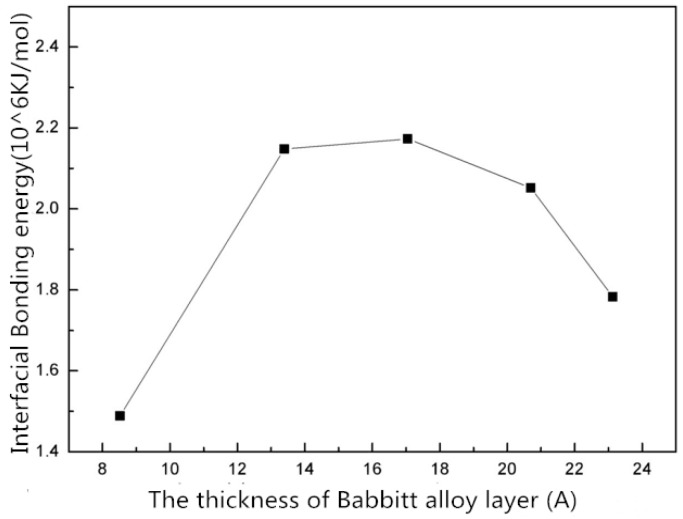
The interface structure energy with different Babbitt thicknesses.

The energy in the Table 5 is graphically represented in Figure 6 and Figure 7.

As shown in Figure 6, the total energy of the interface structure increases with the increasing thickness of the Babbitt layer. The energy of the steel layer in the interface structure varies little with the changing thickness of the Babbitt layer. As can be seen from Figure 7, the interfacial energy first increases with the increasing thickness of the Babbitt layer and then decreases. When the thickness of the Babbitt alloy layer in interface structure is 17.043 Å, the maximum interface bonding energy can reach 2.173 × 10^6^ kJ/mol.

## 5. Conclusions

(1) The bonding interface between Babbitt alloy and steel body with or without a Sn layer was simulated using molecular dynamics, and the bonding energies of the bonding interface with or without a Sn layer were obtained respectively. Through comparison, it is concluded that the bonding interface of Babbitt and steel with a Sn layer is better than that without a Sn layer, and the interface bonding energy with a Sn layer is larger than that without a Sn layer by 10%.
(2) From the energy compositions of different interfaces, the main forces acting on the interface structure of the bushing are Van Der Waals’ force and electrostatic force, which play a major role in the interactions between interfaces, and the constraining energy can be neglected. The main factor affecting the interfacial bonding energy is non-bonding energy.
(3) The interface structures of the Babbitt layer with different thicknesses were quantitatively analyzed using a molecular dynamics simulation. The bonding energy of the interface changes with the thickness of Babbitt in the interface structure. When the thickness of the Babbitt alloy layer is 17.043 Å, the bonding performance of the interface is optimum.

## Figures and Tables

**Table 1:** The parameters of material cell structure.

Material		Steel Body	Sn	Babbitt Metal
Space Group		CMC21	FD-3M	P63/MMC
Syngony		Orthorhombic system	Orthorhombic system	Hexagonal crystal system
Cell length	a (Å)	10.108	6.491	4.217
	b (Å)	7.998	6.491	4.217
	c (Å)	7.546	6.491	5.120
Cell angle	α	90	90	90
	β	90	90	90
	γ	90	90	120

**Table 2:** Energies of structures with different interfaces.

Interface Type	$E_{\mathrm{total}}$ (kcal/mol)	$E_{\mathrm{steel}}$ (kcal/mol)	$E_{\mathrm{Babbitt}}$ (kcal/mol)	$E_{\mathrm{bonding}}$ (kcal/mol)
Without Sn interface	4,776,554.60	3,291,967.51	964,648.17	519,938.92
With Sn interface	5,445,204.16	3,720,407.89	1,154,367.45	570,428.82

**Table 3:** Interfacial energy composition.

Interface Type	Non-Bonding Energy (kcal/mol)		Bonding Energy (kcal/mol)
	Van Der Waals Energy	Electrostatic Energy	
Without Sn interface	141,536.66	4,635,308.39	−290.45
With Sn interface	206,368.67	5,239,210.25	−374.76

**Table 4:** The energy of the interface structure with different Babbitt thicknessess.

Babbitt Layer Thickness (Å)	8.521	13.391	17.043	20.695	23.13
Total interfacial energy $E_{\mathrm{total}}$ (kcal/mol)	4,114,386.435	4,482,626.633	4,776,554.600	5,079,386.406	5,165,204.487
Babbitt layer energy $E_{\mathrm{Babbitt}}$ (kcal/mol)	411,785.390	697,262.041	964,648.171	1,279,625.574	1,441,594.477
Steel body layer energy $E_{\mathrm{Steel}}$ (kcal/mol)	3,344,574.554	3,271,396.980	3,291,967.512	3,308,934.658	3,297,101.787
Interface Bonding energy $E_{\mathrm{bonding}}$ (kcal/mol)	358,026.491	513,967.613	519,938.918	490,826.174	426,508.225

**Table 5:** Interfacial bonding energy with different thicknessess of Babbitt.

Babbitt Layer Thickness (Å)	8.521	13.391	17.043	20.695	23.13
Interface Bonding energy $E_{\mathrm{bonding}}$ (KJ/mol)	$1.498\times 10^{6}$	$2.148\times 10^{6}$	$2.173\times 10^{6}$	$2.052\times 10^{6}$	$1.783\times 10^{6}$
